# Assessing the feasibility, acceptability and potential effectiveness of Dignity Therapy for people with advanced cancer referred to a hospital-based palliative care team: Study protocol

**DOI:** 10.1186/1472-684X-8-5

**Published:** 2009-05-16

**Authors:** Sue Hall, Polly Edmonds, Richard Harding, Harvey Chochinov, Irene J Higginson

**Affiliations:** 1 Department of Palliative Care, Policy & Rehabilitation, King's College London, Weston Education Centre, Cutcombe Road, London SE5 9RJ, UK; 2 Palliative Care Team, King's College Hospital NHS Foundation Trust, Denmark Hill, London SE5 9PJ, UK; 3 Manitoba Palliative Care Research Unit, CancerCare Manitoba, Department of Psychiatry, University of Manitoba, 3017-675 McDermot, Winnipeg, MB R3E 0V9, Canada

## Abstract

**Background:**

Loss of dignity for people with advanced cancer is associated with high levels of psychological and spiritual distress and the loss of the will to live. Dignity Therapy is a brief psychotherapy, which has been developed to help promote dignity and reduce distress. It comprises a recorded interview, which is transcribed, edited then returned to the patient, who can bequeath it to people of their choosing. Piloting in Canada, Australia and the USA, has suggested that Dignity Therapy is beneficial to people with advanced cancer and their families. The aims of this study are to assess the feasibility, acceptability and potential effectiveness of Dignity Therapy to reduce psychological and spiritual distress in people with advanced cancer who have been referred to hospital-based palliative care teams in the UK, and to pilot the methods for a Phase III RCT.

**Design:**

A randomised controlled open-label trial. Forty patients with advanced cancer are randomly allocated to one of two groups: (i) Intervention (Dignity Therapy offered in addition to any standard care), and (ii) Control group (standard care). Recipients of the 'generativity' documents are asked their views on taking part in the study and the therapy. Both quantitative and qualitative outcomes are assessed in face-to-face interviews at baseline and at approximately one and four weeks after the intervention (equivalent in the control group). The primary outcome is patients' sense of dignity (potential effectiveness) assessed by the Patient Dignity Inventory. Secondary outcomes for patients include distress, hopefulness and quality of life. In view of the relatively small sample size, quantitative analyses are mainly descriptive. The qualitative analysis uses the Framework method.

**Discussion:**

Dignity Therapy is brief, can be delivered at the bedside and may help both patients and their families. This detailed exploratory research shows if it is feasible to offer Dignity Therapy to patients with advanced cancer, many of whom are likely to be in the terminal stage of their illness, whether it is acceptable to them and their families, if it is likely to be effective, and determine whether a Phase III RCT is desirable.

**Trial registration:**

Current Controlled Clinical Trials: ISRCTN29868352

## Background

There has been much progress in the assessment and treatment of the somatic symptoms of people with advanced cancer, however less is known about assessing and treating psychosocial, existential and spiritual issues. Loss of dignity for people with advanced cancer is associated with high levels of psychological and spiritual distress and the loss of the will to live[1]. For some people, a sense that nothing of one's life will be transcendent of death was associated with loss of dignity, and many felt that maintaining dignity was highly dependent on how they perceived themselves to be seen by others. It is therefore crucial to develop and evaluate interventions to address these needs. An empirically based model of dignity in the terminally ill has been developed in Canada from interviews with 50 people with advanced cancer focussing on what supports and what undermines their dignity [2,3]. The model comprises three major categories (illness-related concerns, dignity conserving repertoire, social aspects of the illness experience), which refer to broad issues that determine how individuals experience a sense of dignity as death approaches. Each of the three categories has several themes and sub-themes. The model provides a theoretical framework, which helps understanding of how people with advanced cancer may experience a loss of dignity and provides the theoretical base for a therapeutic intervention: dignity therapy[4].

Dignity Therapy is a brief, individualized intervention to increase the sense of purpose, meaning and worth and reduce spiritual and psychological suffering for people with advanced cancer. The therapy can be delivered at the bedside by health care professionals (after brief training). The therapist conducts an interview, which is based on the dignity model[2]. Individuals are offered the opportunity to address aspects of life they feel most important, such as recounting parts of their life they feel proudest of, things they feel are or were most meaningful, the personal history they would most want remembered, or advice to their family and friends. Interviews last between 30 and 60 minutes. They are tape recorded, transcribed, edited, (see methods for details of editing procedure) and quickly returned to the patient to share with people of their choosing. These 'generativity' documents allow people to leave behind something lasting. An important feature of the therapy is that it also has the potential to help friends and relatives in their bereavement[5].

A preliminary evaluation of Dignity Therapy conducted with patients with advanced cancer in Canada and Australia have produced positive findings for patients[4] and their families[5]. Ninety-one percent of participants reported being satisfied with dignity therapy; 76% reported a heightened sense of dignity; 68% reported an increased sense of purpose; 67% reported a heightened sense of meaning; 47% reported an increased will to live; and 81% reported that it had been, or would be of help to their family. Randomised controlled trials (RCTs) of Dignity Therapy for hospice patients are underway in Canada, Australia and the USA, assessing outcomes immediately after the intervention. However, the longer term impact of the intervention on patients is not being assessed. Furthermore, since responses to Dignity Therapy may be influenced by a range of important social and cultural factors, the results of these trials may not be generalisable to the UK. There are a number of potential moderators of the impact of intervention (e.g. ethnicity, age, cognitive acuity, stage of illness, baseline levels of distress). Answering these questions would require a randomised controlled trial in the UK. There are a number of methodological and ethical issues concerning conducting RCTs of patients with advanced cancer[6], including concerns about randomisation, loss to follow-up and appropriate user involvement in the research.

In-depth piloting, which includes an exploration of the feasibility of delivering an intervention and attention to the context in which interventions take place, as planned here, is recommended in the new Medical Research guidance for developing and evaluating complex interventions[7]. As recommended in the MRC framework, we are also testing our proposed outcome measures. The results will inform the design of a Phase III RCT.

## Aims and objectives

The aims of the study are to assess the feasibility, acceptability and potential effectiveness of Dignity Therapy to reduce psychological and spiritual distress in people with advanced cancer who have been referred to hospital-based palliative care teams. The specific objectives are to:

a) Determine whether Dignity Therapy is likely to increase peoples' sense of dignity or reduce psychological or spiritual distress.

b) Determine whether it is feasible to provide Dignity Therapy in this setting.

c) Determine whether Dignity Therapy is acceptable to patients and their families.

d) Pilot methods for a larger (Phase III) RCT (e.g. recruitment, randomization, follow-up, suitability of measures, effect size).

## Methods

### Study design

A Phase II open-label RCT comprising two groups: (i) Intervention (Dignity Therapy offered in addition to standard care), and (ii) Control group (standard care). Standard care comprises assessment by a multi-professional palliative care team, including nurses, a psychosocial worker and doctors trained in providing psychosocial support. Consenting participants have been randomly allocated to one of these two groups after baseline (T1) measures have been collected.

### Randomization

Randomization was conducted by an independent statistician. Treatment allocation (Dignity Therapy or control) was performed by block randomization with a fixed block size of two. Allocation concealment is facilitated by using sequentially numbered opaque sealed envelopes for consecutive and eligible participants. To reduce the risk of bias, the research assistant opens the next envelope in the presence of the patient to ascertain which group the patient has been allocated to after baseline measures have been collected from participants.

### Ethical Approval

This study has been approved by The King's College Hospital Research Ethics Committee (10/11/2008, ref: 08/H0808/155), and meets the requirements of the local Research Governance Framework.

### Participants

The sample will comprise 40 adults with advanced cancer who have been referred to hospital-based palliative care teams working in two NHS Trusts, and who are well enough to participate in a protocol lasting two weeks. For this pilot study, we are obtaining in-depth information on taking part in the study and receiving the therapy from a relatively small sample rather than planning to detect significant group differences. One of the aims of the study is to estimate the effect size for an RCT.

#### Inclusion criteria

Patients with a diagnosis of cancer aged 18 years or over are included. We are not selecting people on the basis of prognosis or stage of disease, however, since patients are receiving palliative care, most are expected to have advanced cancer. Participants are not screened for spiritual or psychological distress, or loss of dignity, however, these are assessed at baseline to explore the potential moderating effects of these variables on the impact of the intervention.

#### Exclusion criteria

Patients are excluded if the palliative care team feel they are unable to take part in a protocol lasting two weeks (the time taken to collect baseline measures and complete the intervention), or if they are unable to provide informed consent either due to cognitive problems or the severity of their illness, or because they are unable to understand English. Patients with moderate or severe cognitive impairment are excluded since their 'generativity' documents are likely to reflect a fractured sense of self, which could be distressing to them and recipients of these documents.

A close family member or friend (for participants in both groups) is invited into the study to obtain their views on taking part in the study and the therapy (intervention group only). These are usually the recipients of the 'generativity' documents, however, any additional recipients will also be invited to take part to obtain their views on the therapy.

### The intervention

The therapy is delivered by a palliative care nurse who has been trained in Dignity Therapy by Harvey Chochinov (who developed it). Training included the theoretical basis for the intervention, demonstrations of Dignity Therapy, a detailed overview of the therapy manual, editing the therapy documents and working with patients to produce a document that will be helpful for them and its recipients.

A standard framework of questions used in the therapy (Appendix 1) is given to patients in the intervention group (after randomization) to give them the opportunity to think about their responses before the session. The question framework provides a flexible guide for the therapist to shape the interview, based on patients' level of interest and responses. The therapist follows the patients' cues, helping them to structure and organise their thoughts, for example, by asking questions about time sequences, how events are causally related to each other and facilitating the disclosure of thoughts, feelings and memories. If a patient prefers a friend or relative to be present during the dignity therapy session, they can either listen or join in. Patients sometimes find they need help to recall details. This strategy has proved to be very helpful for some patients in the piloting of Dignity Therapy in Canada, Australia & USA. The role of the person sitting in will be agreed with the patient beforehand, and the therapist will ensure that the discussion is led by the patients rather than their friends or relatives.

These interviews are tape-recorded, quickly transcribed verbatim then shaped into a narrative using a formatted editing process. This includes clarifications to eliminate colloquialisms, non-starters and irrelevant sections (such as interruptions), chronological corrections, tagging and editing any content that might inflict significant harm on recipients of the document (after discussion with the patient) and finding a suitable ending for the document which is appropriate to the patients' overall message. Another session is arranged for the therapist to read the edited transcript to the participants, who are invited to make any editorial suggestions, including identifying errors of omission or commission. Once these 'generativity' documents are finalised they become the property of the patient, who can share it with anyone they choose, whenever they choose. We give them as many copies as they wish. At the start of the therapy, we ask patients who they intend to leave the 'generativity' document to, as this enables the therapist to guide the patient to cover the areas most relevant to the intended recipient(s). However, patients can change their mind about this at any time. They may decide not to give their 'generativity' document to anyone, or give it to other recipients. We do not exclude patients who do not wish to pass on their document. If they do not wish to bequeath it to anyone they can either destroy it or leave it with their other personal documents such as their will, diaries and important letters. The therapist discusses this with any patients who decide not to bequeath their document.

Since participants' conditions can fluctuate rapidly, the timing of the contacts can be relaxed and meetings rescheduled. If a participant's condition deteriorates, meetings are rescheduled up to three times before sensitively withdrawing them from the study. The therapist makes detailed notes of her experiences of giving each intervention and any deviations from the protocol. One in three therapy transcripts is randomly selected for review by the principal investigators. A quality assurance protocol has been developed to assess adherence to the Dignity Therapy protocol and deviations from the protocol will be reported as part of the feasibility study. This includes whether or not the therapist was respectful and asked the questions from the Dignity Therapy protocol appropriately, and whether or not the editing process was carried out in accordance to the protocol.

### Control group

Patients in the control group have at least three interviews with a research assistant. Completing the measures and taking part in the interview gives them an opportunity to talk about their feelings. The extent to which they feel that this is therapeutic is explored in the follow-up interviews.

### Recruitment procedure

**Figure 1:**
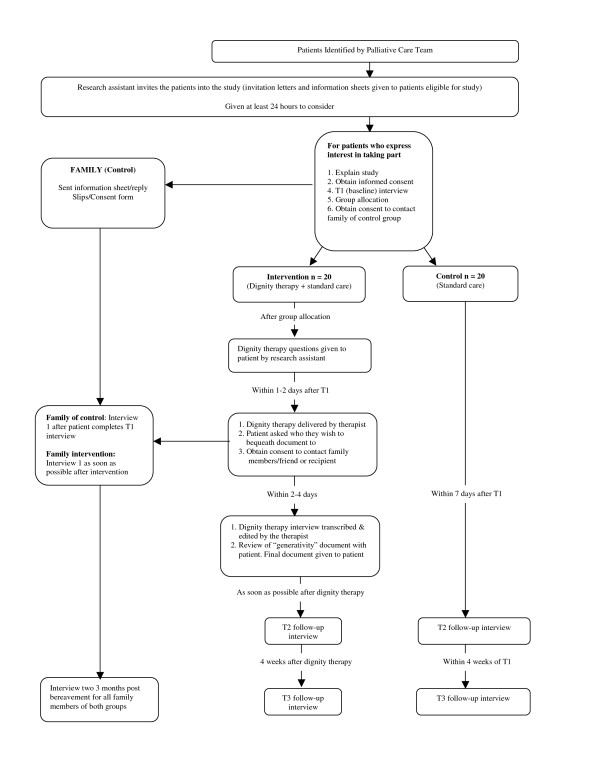
**Recruitment and follow-up procedure**.

The recruitment and follow-up procedure is shown in Figure 1. Potential participants are identified by palliative care teams from their lists. For patients recruited from one NHS Trust, these are in-patients or those visiting out-patient clinics. The research assistant gives potential participants an invitation letter and patient information sheet after being introduced by the palliative care team. The research assistant visits patients at least 24 hours later to find out if they are interested in taking part. From the other Trust we are recruiting patients cared for in the community. The clinical nurse specialist visiting the patient will give them the invitation letter and patient information sheet. They will be asked to send a reply slip to the research team or to notify the Clinical Nurse Specialist of their decision to take part or not. Patients are encouraged to discuss involvement in the study with family or friends before deciding whether or not to take part.

Before consent is taken from patients who have expressed an interest in taking part, the research assistant checks they have fully understood the patient information sheet before obtaining written consent from those who are willing to take part. As a final check patients are screened with the Blessed Orientation Memory Concentration Test [8] to assess cognitive functioning after the consent procedure. It has been suggested by Chochinov (personal communication) that Dignity Therapy is not suitable for patients with a score equal to or greater than 15. In such cases patients are excluded. This is done sensitively: the research assistant spends some time chatting with them about neutral topics. This approach has worked successfully in a previous study[9]. The proportion of patients excluded at this stage will be reported. Oncologists in the Trusts have been informed of the study and General Practitioners will be informed of their patients' involvement.

Following the consent procedure, participants are asked to complete the baseline measures. They are then randomized. Those in the intervention group are given the framework of therapy questions (Appendix 1) so that they have an opportunity to think about their answers before the therapy interview. The therapist arranges to conduct the dignity therapy interview as soon as possible after recruitment (usually within two to three days), in the location most suited to the patient and in the most private area available if they are in hospital. Since the condition of participants could fluctuate rapidly, the timing of the contacts are sometimes adjusted and meetings rescheduled. If a participant's condition deteriorates, meetings are rescheduled up to three times before sensitively excusing them from the study. This, and the feasibility of following up with people if they leave hospital, will also be reported and taken into account when developing the RCT. Participants in the control group are reminded that they will still have the opportunity to talk to the researcher about how they are feeling and about their views on taking part in the study.

At recruitment the research assistant asks participants to nominate a close family member or friend who is invited to take part in a telephone interview. For patients in the intervention group the nominated family member or friend is often the recipient of the 'generativity' document, however, if the participant mentions other recipients they will also be invited for interview. Participants are not excluded if they do not nominate a family member or friend. We contact the palliative care teams to determine when a participant has died and conduct interviews three months post bereavement with all nominated family and friends and any additional recipients of the document.

### Outcomes

Both quantitative and qualitative outcomes are assessed (Table 1). These are collected from patients by face-to-face interviews at two time-points: approximately one (T2) and four weeks (T 3) after the dignity document has been completed, and the equivalent in the control group. This longer-term follow-up has not been included in the previous evaluation of Dignity Therapy, or in the current trials being conducted by Chochinov and colleagues. However, we believe it is important to evaluate the longer-term impact of the intervention. The feasibility of doing this is explored as part of this feasibility study. Quantitative measures have been validated and are fairly quick and easy to use. Qualitative interview schedules have been developed for the study.

**Table 1:** Measures for each time point

	**Patients**		**Family/friends**	
	Intervention	Control	Intervention	Control
**At patient recruitment**				
Patient characteristics	x	x		
Patient Dignity Inventory^10^	x	x		
Herth Hope Index^13^	x	x		
Hospital Anxiety & Depression Scale^11^	x	x		
Distress Thermometer^12^	x	x		
EQ-5D^14 ^(Quality of life)	x	x		
Two item measure of quality of Life	x	x		
Palliative Care Outcome Scale^15^	x	x		
**As soon as possible post intervention**				
Acceptability of therapy/study (interviews)	x	x	x	x
Patient Dignity Inventory^10^	x	x		
Herth Hope Index^13^	x	x		
Hospital Anxiety & Depression Scale^11^	x	x		
Distress Thermometer^12^	x	x		
EQ-5D^14 ^(Quality of life)	x	x		
Two item measure of quality of Life	x	x		
Palliative Care Outcome Scale^15^	x	x		
**One month post intervention**				
Acceptability of therapy/study	x	x		
Patient Dignity Inventory^10^	x	x		
Herth Hope Index^13^	x	x		
Hospital Anxiety & Depression Scale^11^	x	x		
Distress Thermometer^12^	x	x		
EQ-5D^14 ^(Quality of life)	x	x		
Two item measure of quality of Life	x	x		
Palliative Care Outcome Scale^15^	x	x		
Three months post bereavement*				
Hospital Anxiety & Depression Scale^11^			x	x
Complicated Grief assessment^20^			x	x
Acceptability of therapy/study			x	x
Palliative Care Outcome Scale^15^			x	x

* It is likely that it will not be possible to collect post bereavement outcomes from all the recipients of the legacy documents within the relatively short timeframe of this pilot study. However, in depth qualitative data on their views of the therapy, taking part in the study and completing the outcome measures will be obtained from those who are interviewed.

#### Main outcome for patients

The primary outcome is patients' sense of dignity (potential effectiveness). This is assessed at baseline, (T1) and at T2 and T3 follow-ups, using the Patient Dignity Inventory[10]. This measure evolved directly from the dignity model, therefore, questions correspond to each of the model themes and sub-themes, including: physical, psychosocial, existential and spiritual domains of concern or distress. This measure has been validated in Canada and has been shown to have excellent face, internal, test-retest and concurrent validity.

#### Secondary outcomes for patients

Potential effectiveness is also assessed using: the Hospital Anxiety and Depression scale[11] and the distress thermometer [12] (psychological distress); the Herth Hope Index[13] (hopefulness); the EQ-5D[14], the POS (Palliative Care Outcomes) [15] and a two item measure used by Harvey Chochinov in the current trials of Dignity Therapy (both quality of life). To assess feasibility of delivering Dignity Therapy in this setting, time taken to organize and conduct the Dignity Therapy sessions, transcribe and edit narratives, deviations from the therapy protocol and uncompleted interventions and the reasons, and the therapist's perceptions of competence as a result of training are recorded. To assess the acceptability of Dignity Therapy we are conducting semi-structured interviews with participants in the intervention group to obtain their views on the intervention. The therapist is recording her experiences of delivering the therapy and her observations of patient's responses during and after the therapy. As suggested by Ferrell[16], we are reporting case studies of any difficult cases.

#### Demographic measures

Demographic information is collected, including: cognitive functioning (using the Blessed Orientation Memory Concentration test[8]), co-morbidity (using the Comorbidity Index and Scores of Charlson[17]), performance status (using Karnosfsky scores[18], and ability to perform activities of daily living (Barthel scores[19]), age, gender, ethnic group.

#### Outcome measures for family/friends/recipients of 'generativity' documents

In completion of the therapy, semi-structured telephone interviews are conducted with patients' family/friends and other recipients of the 'generativity' documents to obtain their perceptions of the impact of the intervention, on themselves and on the patient (intervention group), and their views on taking part in the study (both groups). For patients who die during the data collection period of the study, follow-up telephone interviews are conducted with family/friends/recipients of the 'generativity' documents at three months post bereavement. As in the initial interview, these cover their perceptions of the impact of the intervention, on themselves and on the patient, and their views on taking part in the study. They are also asked to complete the Complicated Grief Assessment[20], which is used to screen for complicated grief reactions and the Hospital Anxiety and Depression Scale [11]. The questionnaire is mailed to them after the telephone interview along with a pre-paid envelope in which to return it. Their age, gender, ethnic group and relationship to patients are also recorded.

#### Piloting methods for a Phase III trial

Time taken to obtain informed consent and collect outcomes, exclusions, recruitment and drop-out rates (patients and their family and friends) are recorded. In addition their views on taking part in the study (e.g. being randomized) are sought in qualitative interviews, and their views on completing the outcome measures are recorded when they are completing them. Any problems with completing measures will be reported, including missing data.

### Analyses

#### Quantitative data

In view of the relatively small sample size, analyses will be mainly descriptive, however, between and within participant comparisons of outcomes will be conducted and the appropriate effect size estimates reported. Either parametric or non-parametric tests will be used, depending on the distribution of the data. The intervention and control group will be compared on the main outcome (a sense of dignity) and secondary outcomes (distress, hopefulness, quality of life and palliative care outcomes). We will also compare Time 2 and Time 3 follow-up with baseline for both groups on these measures, using a paired test. The two groups will also be compared on demographic characteristics and baseline measures. We will also report recruitment rates and compare drop-out rates and missing data in the two groups.

#### Qualitative data

We are using the Framework method of analysis [21]. Analyses are both deductive (from pre-set aims and objectives) and inductive (arising from participants views). This method tends to be more structured than some other methods of qualitative analysis and the process more explicit and more informed by a priori questions. It is designed so that it can be more easily understood and assessed by people other than the analyst, such as funding bodies, policy makers and participants. Throughout the analytical process we use strategies to maximise credibility, criticality, and authenticity[22]. The QSR NVivo software package is used to manage the qualitative data.

## Discussion

There is a dearth of interventions to reduce psychological and spiritual distress for people with advanced cancer. Dignity Therapy is brief, can be done at the bedside and aims to help both patients and their families. The proposed detailed exploratory research will show if it is feasible to offer Dignity Therapy to patients with advanced cancer who have been referred to hospital-based palliative care teams, whether it is acceptable to them and their families, if it is likely to be effective, and determine whether a Phase III RCT is needed. As suggested by Ferrell [16], we plan to report the therapists experiences of delivering the therapy, any difficult cases and how they dealt with them, for example those who had intense regrets about their lives or difficult conflicts to resolve. If the trial shows Dignity Therapy is effective, it could prove to be a relatively low cost intervention which could be offered routinely by trained health care professionals and chaplains.

## Competing interests

The authors declare that they have no competing interests.

## Authors' contributions

SH led the drafting of this paper and development of the protocol. HC developed Dignity Therapy. SH & IJH co-conceived the study. All authors co-applied for funding and contributed to the development of the protocol and the final draft of this paper.

## Appendix 1 framework of questions used in Dignity Therapy

• Tell me a little about your life history; particularly the parts that you either remember most, or think are the most important? Another way of putting this, which may elicit answers from some patients, is to ask, when did you feel most alive?

• Are their particular things that you would want your family to know about you, and are their particular things you would want them to remember?

• What are the most important roles you have played in your life (family roles, vocational roles, community service roles, etc)? Why were they so important to you, and what do you think you accomplished within those roles?

• What are your most important accomplishments, and what do you feel most proud of?

• Are there particular things that you feel still need to be said to your loved ones, or things that you would want to take the time to say once again?

• What are your hopes and dreams for your loved ones?

• What have you learned about life that you would want to pass along to others? What advise or words of guidance would you wish to pass along to your [son, daughter, husband, wife, parents, other(s)]?

• Are their words or perhaps even instructions you would like to offer your family, in order to provide them with comfort or solace?

• In creating this permanent record, are their other things that you would like included?

## Pre-publication history

The pre-publication history for this paper can be accessed here:


